# Transvaginal laparoscopic right colectomy for colon neoplasia

**DOI:** 10.1093/gastro/goz059

**Published:** 2019-11-09

**Authors:** Yi Xiao, Lai Xu, Jun-Ji Zhang, Pei-Ran Xu

**Affiliations:** 1 Department of Surgery, Peking Union Medical College Hospital, Chinese Academy of Medical Sciences, Beijing, P. R. China; 2 Department of Obstetrics & Gynecology, Peking Union Medical College Hospital, Chinese Academy of Medical Sciences, Beijing, P. R. China

## Introduction

The laparoscopic technique has been used for colorectal surgery for decades. Minimally invasive surgery is executed differently in operations on the right colon, left colon, and the rectum [1]. Sometimes, the abdominal incision is critical for rectal surgery, which can be used for extraction of the resected specimen and for creation of the diverting stoma. However, for right or left colectomy, once the anastomosis is performed intra-corporeally, then the specimen can be extracted through any incision (abdominal wall or vaginal) to enhance post-operative recovery.

The natural-orifice transluminal endoscopic surgery (NOTES) approach has been used in operations on the colon and rectum to minimize abdominal trauma, as, for example, in transanal total mesorectal excision (taTME) [2], laparoscopic colectomies with transanal [3] or transvaginal specimen extraction [4]. Up to now, transabdominal assistant with the laparoscopic technique is critical for accomplishing colorectal NOTES operations, so it is called the hybrid NOTES technique [5, 6]. Pure NOTES has not been reported for colonic resection. Recently, we successfully performed a laparoscopic right colectomy via the transvaginal approach. Here we describe the procedure.

## Surgical procedures

A 65-year-old woman presented with complaints of diarrhea, abdominal distention, and rectal tenesmus for >1 month; she had no nausea, fever, hematochezia, or melena. She had reached menopause 14 years ago and had a body mass index of 28.5 kg/m^2^. Laboratory tests (blood routine examination, serum tumor markers, serum biochemistry) were normal. Abdominal ultrasound revealed a hypoechoic mass to the right of the umbilicus. Colonoscopy showed a 4-cm-diameter lobulated mass in the ascending colon. Biopsy specimen sent for pathological examination showed high-grade intraepithelial neoplasia. Chest-abdomen-pelvis contrast-enhanced CT scan revealed thickened wall and luminal stenosis of the ascending colon. No distant metastases were detected.

**Figure 1.**
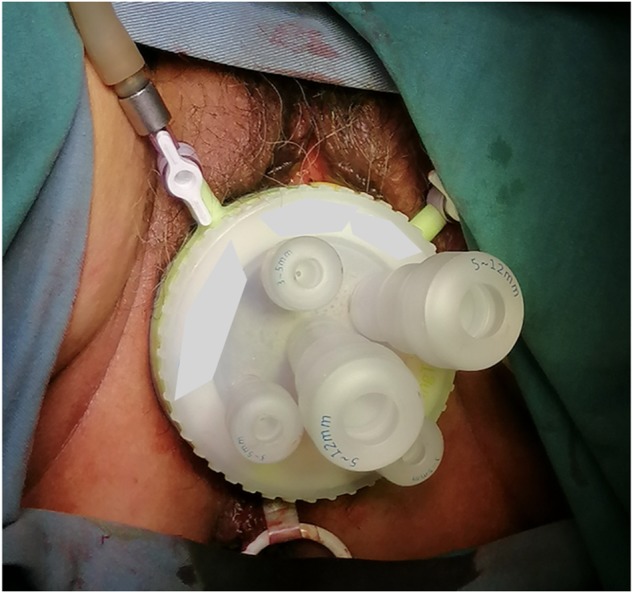
The transvaginal single-port device

Under general anesthesia, the patient was placed in the lithotomy position. The anterior vaginal fornix was opened and a single-port laparoscopic device (Single Port, ACCESS SYSTEM, HTKD Medical^TM^) was placed (Figure 1). Long laparoscopic instruments, with a length of 43 cm, were introduced. Pneumoperitoneum was established at the level of 14 mmHg and the small intestine was shifted to the left side by rolling over the patient to right-side elevated. The caudal approach was preferred and the peritoneum distal to the cecum was incised by a harmonic scalpel. The dissection plane was extended in the retrocolic space by retracting the cecum upward and cranially. The ileum was transected by stapler (articulating endoscopic linear cutter, PSEE60A, Echelon Flex^TM^, ETHICON, Chihuahua, Mexico) at 10 cm proximal to the cecum and then its mesentery was cut toward centrally.

**Figure 2.**
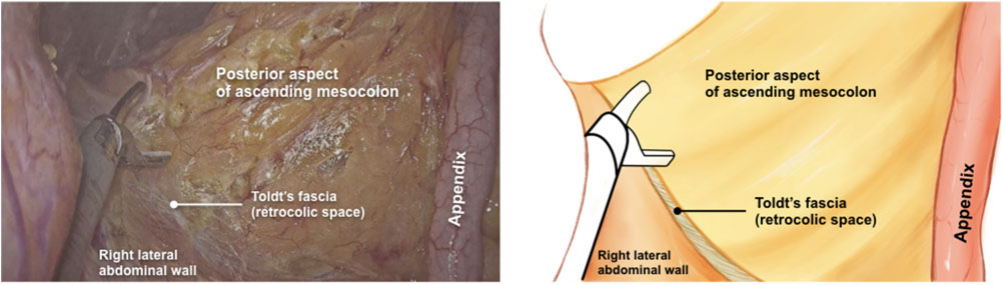
The right retrocolic space was identified and the appendix as well as the cecum and its mesentery was pulled upward and cranially

**Figure 3.**
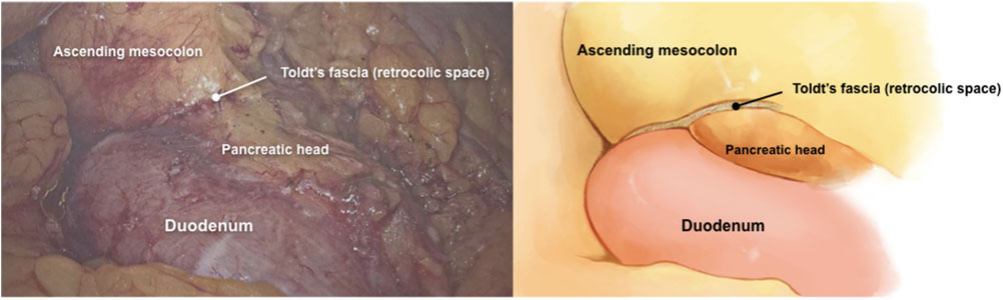
The anterior surface of the duodenum and the pancreatic head were exposed after dissecting along the retrocolic space

**Figure 4.**
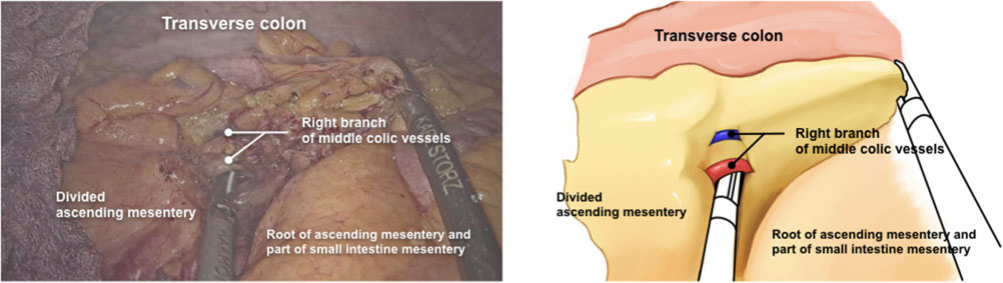
The right branches of the middle colic vessels and the superior right colic vein were divided by the harmonic scalpel

The ileocolic vessels were clamped and divided near their origin from the superior mesenteric artery and vein. The retrocolic space was then dissected cranially up to the hepatic flexure (Figure 2), laterally up to the abdominal wall, and the anterior surface of the duodenum and the pancreatic head were exposed (Figure 3). The right branches of the middle colic vessels and the superior right colic vein were divided by the harmonic scalpel (Figure 4). The greater omentum was stripped down from the stomach at the left one-third of the transverse colon, and the transverse colon and its mesentery were divided by stapler and scalpel separately at the right one-third of the transverse colon. The specimen was removed en bloc via the vaginal incision. A side-to-side overlap ileocolic anastomosis was made using an articulating endoscopic linear cutter. The anastomotic common orifice was closed by 3/0 running seromuscular sutures, with absorbable VICRYL sutures. The specimen was extracted through the vagina by the above-mentioned single-port device, with a wound protector. No drainage was placed. The vaginal incision was closed by full-thickness continuous sutures, with Coated VICRYL^TM^*Plus* (VCP358, ETHICON Inc.) sutures. The operation lasted 210 minutes. Total blood loss was 40 mL.

The patient made a fast and full recovery. She was out of bed 16 hours after surgery, passed flatus at 37 hours, and drank water at 88 hours. She was discharged on post-operative Day 6. The post-operative pathology reported a villous tubular adenoma with high-grade intraepithelial neoplasia, with clear resection margins and retrieval of 15 negative lymph nodes.

## Discussion

In the early time of laparoscopic surgery, abdominal incision was indispensable for specimen retraction. As time went on, the NOTES technique, as well as transvaginal specimen retraction and single-port surgery, was introduced. This gradual change in the minimally invasive concept represents a trend of development in surgery. To date, the NOTES technique has not been established in colorectal surgery. The transvaginal approach is mostly used for specimen retraction [4] and should be clearly demarcated for its fully transabdominal laparoscopic resection. Up to now, most right or left colectomy by the NOTES technique have needed one to four transabdominal trocars to assist [5].

This is the first report of right colectomy performed using transvaginal NOTES without transabdominal assistance. During the operation, finding the correct anatomical plane is an essential step. Starting by cutting the peritoneum distal to the cecum might be an easy way to get into the retrocolic space. Besides, we found that it was difficult to transect the mesenteric vessels, mesentery, and then the bowel, in that order, and so we accomplished these procedures in the reverse order.

We hope that the transvaginal approach might be a feasible alternative to transabdominal laparoscopic colectomy. The transvaginal approach for colonic resection, by avoiding abdominal incisions and the associated complications, enhances post-operative recovery. The safety and effectiveness of transvaginal colectomy need to be verified on more patients and need evidential support.

## Funding

None.
